# Predictive and prognostic value of intravoxel incoherent motion (IVIM) MR imaging in patients with advanced cervical cancers undergoing concurrent chemo-radiotherapy

**DOI:** 10.1038/s41598-017-11988-2

**Published:** 2017-09-14

**Authors:** Li Zhu, Huanhuan Wang, Lijing Zhu, Jie Meng, Yan Xu, Baorui Liu, Weibo Chen, Jian He, Zhengyang Zhou, Xiaofeng Yang

**Affiliations:** 1 Department of Radiology, Nanjing Drum Tower Hospital, The Affiliated Hospital of Nanjing University Medical School, Nanjing, 210008 China; 2 The Comprehensive Cancer Centre of Drum Tower Hospital, The Affiliated Hospital of Nanjing University Medical School, Nanjing, 210008 China; 3 Philips Healthcare, Shanghai, China; 4 Department of Radiation Oncology and Winship Cancer Institute, Emory University, Atlanta, Georgia 30322 USA

## Abstract

By using the intravoxel incoherent motion (IVIM) model, the diffusion-related coefficient (D) and the perfusion-related parameter (*f*) can be obtained simultaneously. Here, we explored the application of IVIM MR imaging in predicting long-term prognosis in patients with advanced cervical cancers treated with concurrent chemo-radiotherapy (CCRT). In this study, pelvic MR examinations including an IVIM sequence were performed on 30 women with advanced cervical cancers at three time points (within 2 weeks before, as well as 2 and 4 weeks after, the initiation of CCRT). The performance of tumour size and IVIM-derived parameters in predicting long-term prognosis was evaluated. After a median follow-up of 24 months (range, 10∼34 months), 25/30 (83.33%) patients were alive, and 21/30 (70.00%) remained free of disease. A shrinkage rate of maximum diameter (time point 1 vs. 3) ≥ 58.31% was useful in predicting a good long-term prognosis. The IVIM-derived apparent diffusion coefficient (ADC_IVIM_) value at time point 2 and the ADC_IVIM_ and *f* values at time point 3 also performed well in predicting a good prognosis, with AUC of 0.767, 0.857 and 0.820, respectively. IVIM MR imaging has great potential in predicting long-term prognosis in patients with advanced cervical cancers treated with CCRT.

## Introduction

Cervical cancer is the fourth most common gynaecologic malignancy and the fourth leading cause of cancer mortality in women worldwide. First-line treatments for cervical cancer vary depending on the stage of disease, and concurrent chemo-radiotherapy (CCRT) is considered the standard treatment for patients in advanced stages^1^, in whom residual or recurrent diseases are diagnosed in up to 20∼40%^2^. If early surrogate indicators that correlate with long-term outcome metrics can be determined, better treatment can be scheduled by conducting more intensive follow-ups^3^.

Imaging plays a central role in evaluating the short-term response of cervical cancers treated with CCRT, mostly on the basis of morphological changes with conventional MR imaging, according to the Response Evaluation Criteria in Solid Tumors (RECIST)^4^. Tumour size (both the baseline value as well as its regression) has been widely evaluated as a prognostic index in cervical cancers^5–8^, although its value has been debated. For instance, Harry *et al*. have suggested that the initial tumour size may be more related to the chronological age of the tumour than to intrinsic aggressiveness^9^. Moreover, inflammation, oedema, and capillary hypervascularity associated with radiation changes during the therapy may cause misleading bias in the measurement of residual tumours and make drawing an accurate border difficult^10^.

To address this issue, functional imaging modalities offer more advantages by providing both structural and physiological or even metabolic information for cervical cancers. For instance, dynamic contrast-enhanced (DCE) MR imaging has proven useful in predicting better long-term treatment outcomes in cervical cancers with higher tissue permeability^11^, yet this method involves the use of exogenous gadolinium contrast agents. Diffusion-weighted (DW) imaging can generate apparent diffusion coefficient (ADC) values, whose early change rate after therapy also has predictive potential in cervical cancers treated with CCRT^12–14^.

As an expansion of DW imaging, IVIM MR imaging provides both pure diffusion and perfusion-related incoherent microcirculation information by using an increased number of *b* values^15^. IVIM-generated parameters have proven effective in differentiating cervical cancers from benign lesions and in monitoring therapeutic changes during CCRT^16^. However, the role of IVIM MR imaging in predicting the long-term prognosis of cervical cancers after CCRT has not previously been reported.

Therefore, the purpose of this study was to assess whether IVIM-derived parameters taken before and during early therapy are effective imaging biomarkers in predicting long-term outcomes in patients with advanced cervical cancers treated with CCRT.

## Results

### Predictive value of clinical characteristics of patients and their correlation with IVIM parameters

No significant differences were observed in age between the different groups, with *p* values of 0.986. Moreover, different FIGO stages (below FIGO III versus FIGO III and above) showed no significant correlation with prognosis (*p* = 0.690).

All the IVIM parameters except D* showed a significant positive relationship with different follow-up time points, but the age and FIGO stage of patients had weak associations with the parameters (Table 1).

**Table 1:** Correlation between the clinical characteristics and correlated IVIM parameters in advanced cervical cancer patients.

	Age		FIGO stage		Follow-up time point	
	t	*p*	t	*p*	t	*p*
*f*	−2.520	0.286	1.073	0.014*	4.615	0.000*
D	−1.759	0.331	−0.978	0.082	9.475	0.000*
ADC_IVIM_	−1.388	0.247	−1.166	0.169	12.045	0.000*
D*	−0.917	0.499	−0.679	0.362	0.849	0.339

Note: ADC, apparent diffusion coefficient; *p < 0.05.

### Predictive value of short-term outcome evaluated with RECIST

Five patients classified as CR ultimately had poor prognosis (25.00%, 3 deaths and 2 recurrences), and 6 (60.00%) classified as PR ultimately responded well later (Table 2). Hence, the short-term outcome showed a sensitivity of 71.43% and a specificity of 44.44% in predicting the long-term prognosis.

**Table 2:** Patient characteristics.

Clinical features	Value
Number of patients	30
Mean age (range)	51.1 years (24~76)
FIGO stage:	
II	15 (50.00%)
III	9 (30.00%)
IV	6 (20.00%)
Metastasis:	5 (16.67%)
Retroperitoneal lymph nodes	2 (6.67%)
Rectum	1 (3.33%)
Brain	1 (3.33%)
Supraclavicular lymph nodes	1 (3.33%)
Early treatment outcome:	
CR	20 (66.67%)
PR	10 (33.33%)
Long-term prognosis:	
Complete remission	21(70.00%)
Local recurrence	3(10.00%)
Sustained disease	1(3.33%)
Death	5(16.67%)

FIGO: The International Federation of Gynecology and Obstetrics.

### Predictive values of morphological changes during the CCRT

**Figure 1:**
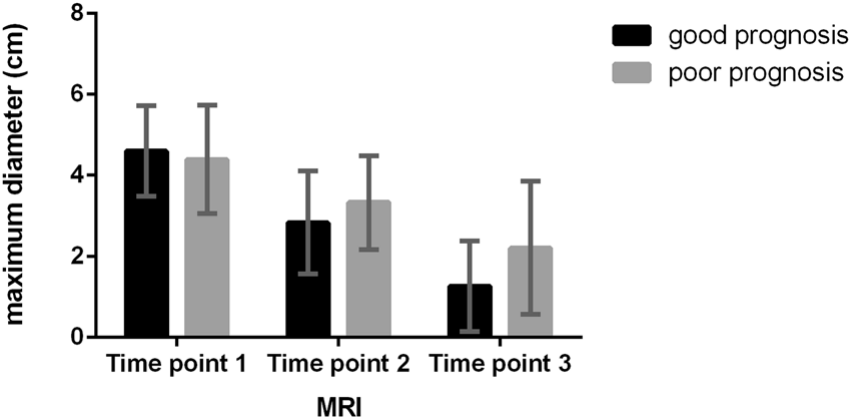
Dynamic changes in maximum diameters of cervical cancers treated with concurrent chemo-radiotherapy (CCRT) in patients with good and poor prognosis, respectively. Significant decreases were observed within both groups, and no significant differences were observed between them.

**Figure 2:**
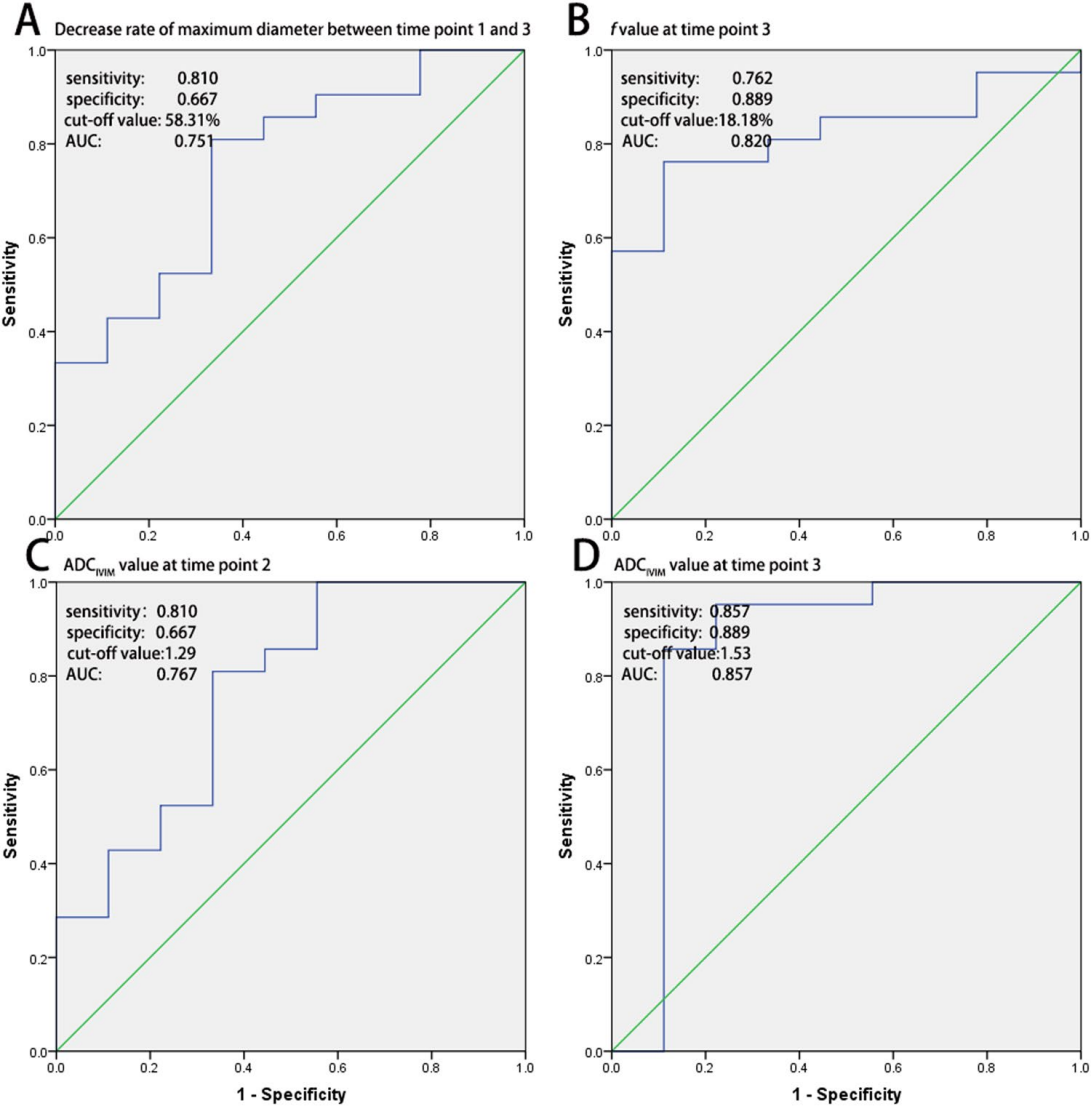
Receiver operating characteristic (ROC) curve of morphological changes, as well as IVIM parameters during CCRT. Decrease rate of maximum diameter between time point 1 and 3 (**A**), *f* value at time point 3 (**B**) and ADC_IVIM_ value at time point 2 (2 weeks after the initiation of CCRT) and 3 (4 weeks after the initiation of CCRT) (**C**,**D**) are useful in predicting the long-term prognosis.

A morphological response was recognized in all patients along with the therapy (Fig. 1), and a greater tumour size decrease (at time point 1 vs. 2 and at time point 1 vs. 3, *p* = 0.027 and 0.018, respectively) was observed in patients with good long-term outcomes than in those with poor outcomes. ROC analysis showed no predictive power in the change rate of maximum diameter (time point 1 vs. 2) as *p* = 0.067, and with a change rate of maximum diameter (time point 1 vs. 3) ≥ 58.31%, the sensitivity and specificity in predicting good outcome were 0.810 and 0.667, respectively (area under ROC curve, AUC = 0.751, *p* = 0.032) (Fig. 2).

### Predictive value of IVIM parameter changes during CCRT

**Figure 3:**
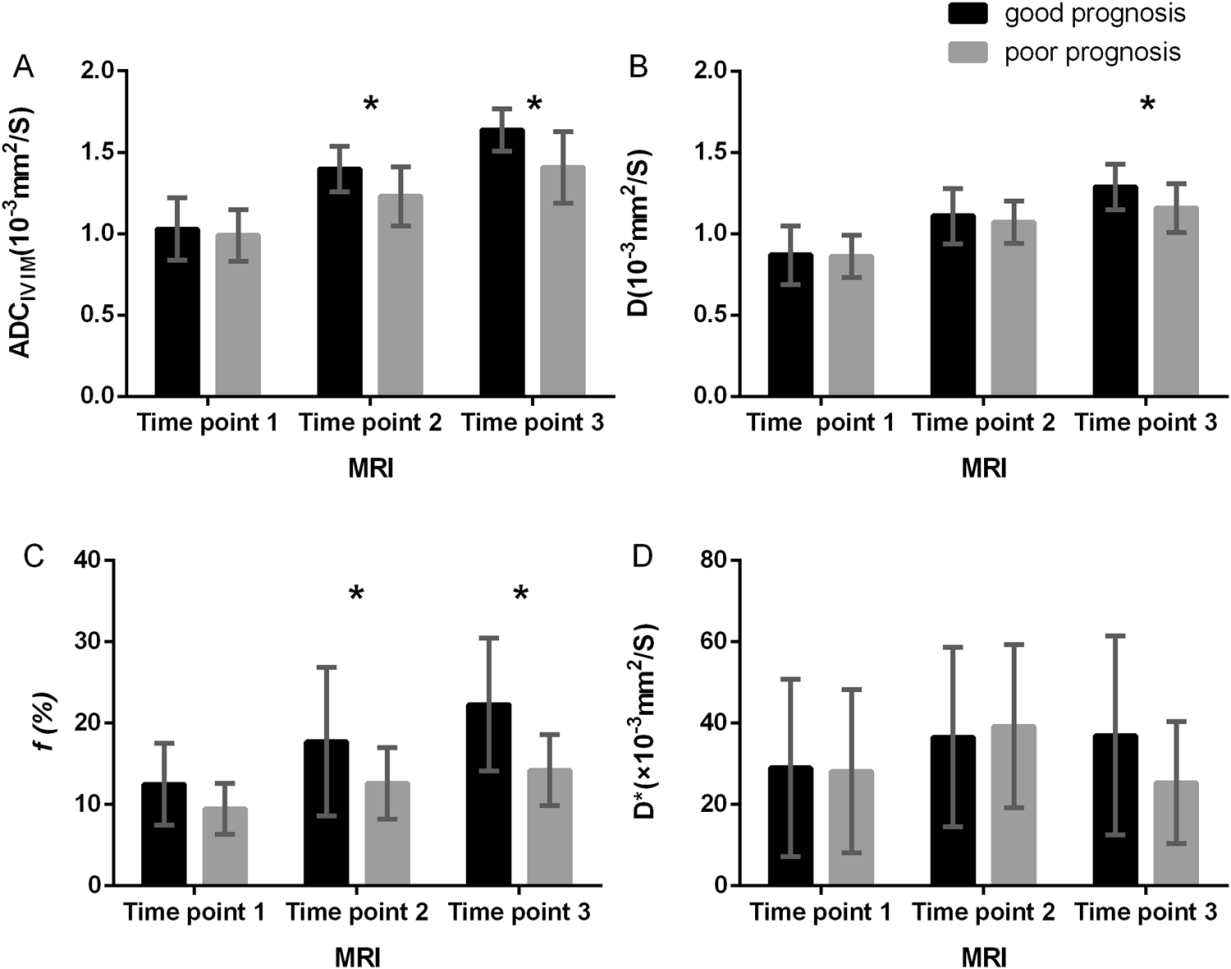
Therapy-induced changes in IVIM parameters along the concurrent chemo-radiotherapy (CCRT). The apparent diffusion coefficient (ADC), D values and *f* value of cervical cancer lesion continued to rise during the therapy course (**A**,**B**,**C**). Significant differences were confirmed in ADC_IVIM_ and *f* values at time point 2, as well as in ADC_IVIM_, *f* and D values at time point 3.

**Figure 4:**
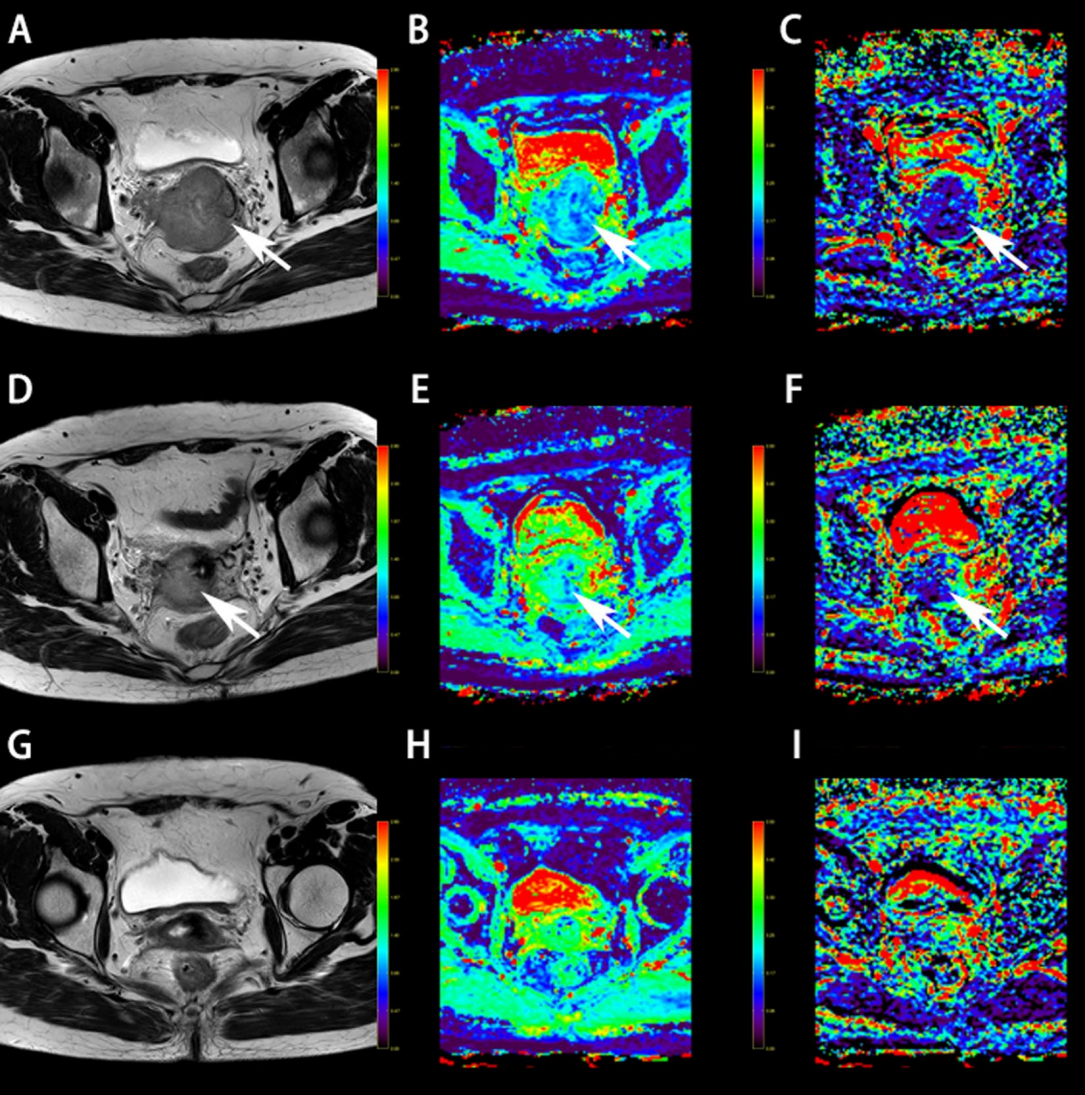
MR images of a 39-year-old female receiving CCRT for advanced cervical cancer (FIGO stage IIB). (**A**,**D**,**G**) Axial T2-weighted images at different time points in therapy (before CCRT, two weeks after initiation of CCRT and one month after initiation of CCRT), showing continuous decrease of the maximum diameter, at 5.07 cm, 3.37 cm and 1.15 cm, respectively; no residual lesion was observed one month after CCRT. (**B**,**E**,**H**) ADC_IVIM_ maps shows an increase in ADC_IVIM_ value during CCRT, as 1.07 × 10^−3^ mm^2^/s; 1.34 × 10^−3^ mm^2^/s and 1.50 × 10^−3^ mm^2^/s, respectively. (**C**,**F**,**I**) *f* maps shows an increasing value during CCRT, as 15.25%, 16.50%, and 27.70%, respectively. After a 28-month follow-up, this patient ultimately had a good prognosis.

All the IVIM-derived parameters except D* values increased during the therapy (Figs 3 and 4). Further, the most dramatic change occurred in the *f* value (change rate, 42.25 ± 54.36%) at time point 2. The ADC_IVIM_ and *f* values at time point 2, as well as the ADC_IVIM_, *f*, and D values at time point 3, differed significantly between patients with good and poor prognosis (all *p* < 0.05), and higher values were observed in patients with good prognosis (Table 3). Further ROC analysis showed the potential of ADC_IVIM_ value at time point 2, as well as the ADC_IVIM_ and *f* value at time point 3, in predicting long-term treatment outcome, with AUC values of 0.767, 0.857 and 0.820, respectively (Fig. 2), whereas no predictive value was found for *f* values at time point 2 (*p* = 0.248) or D values at time point 3 (*p* = 0.054). No change rate index of IVIM parameters during CCRT proved useful in prognostic prediction (Table 4).

**Table 3:** Intravoxel incoherent motion (IVIM)-derived parameters in patients with good and poor prognosis with concurrent chemo-radiotherapy (CCRT).

Parameters	ADC (×10^−3^ mm²/s)		D (×10^−3^ mm²/s)		*f* (%)		D* (×10^−3^ mm²/s)	
Prognosis	Good	Poor	Good	Poor	Good	Poor	Good	Poor
Time point 1	1.03 ± 0.19	0.99 ± 0.16	0.87 ± 0.18	0.86 ± 0.13	12.48 ± 5.03	9.47 ± 3.11	29.00 ± 21.80	28.19 ± 20.08
Time point 2	1.40 ± 0.14	1.23 ± 0.18*	1.11 ± 0.17	1.07 ± 0.13	17.73 ± 9.11	12.59 ± 4.40*	36.54 ± 22.07	39.23 ± 20.08
Time point 3	1.64 ± 0.13	1.41 ± 0.22*	1.29 ± 0.14	1.16 ± 0.15*	22.30 ± 8.16	14.22 ± 4.35*	36.98 ± 24.49	25.40 ± 14.98

Note: ADC, apparent diffusion coefficient; Time point 1, within 2 weeks before CCRT; Time point 2, at the end of the 2nd week; Time point 3, at the end of 4th week during CCRT; *p < 0.05.

**Table 4:** Change rate of Intravoxel incoherent motion (IVIM)-derived parameters in patients with good and poor prognosis.

Change rate	ADC_IVIM_ (%)		D (%)		*f* (%)		D*(%)	
Prognosis	Good	Poor	Good	Poor	Good	Poor	Good	Poor
Time point 1 vs 2	43.74 ± 37.67	25.26 ± 21.61	32.91 ± 43.78	24.47 ± 20.85	46.04 ± 28.13	33.39 ± 28.13	84.08 ± 132.48	100.58 ± 201.97
Time point 1 vs 3	66.56 ± 32.45	45.10 ± 29.36	53.92 ± 34.99	36.61 ± 31.65	90.99 ± 74.94	53.04 ± 31.76	114.05 ± 219.12	23.00 ± 87.63
Time point 2 vs 3	17.51 ± 13.21	16.28 ± 15.94	20.30 ± 28.61	9.39 ± 11.99	44.01 ± 70.79	16.01 ± 20.09	42.29 ± 146.56	−22.60 ± 44.47

Note: ADC, apparent diffusion coefficient; Time point 1, within 2 weeks before CCRT; Time point 2, at the end of the 2nd week; Time point 3, at the end of 4th week during CCRT; *p < 0.05.

### Interobserver agreement in measurement of IVIM parameters

The ICC of ADC_IVIM_, D, *f* and D* values between the two radiologists was 0.840 (95% confidence interval (CI), 0. 756∼0.894; *p* < 0.001), 0.802 (95% CI, 0. 699∼0. 869; *p* < 0.001), 0.886 (95% CI, 0. 827∼0. 925; *p* < 0.001) and 0.291 (95% CI, −0.077∼0.533, *p* = 0.053), respectively.

## Discussion

We demonstrated the feasibility of using IVIM parameters, especially the *f* and ADC_IVIM_ values, to predict long-term prognosis in patients with advanced cervical cancer treated with CCRT, which has never been reported previously.

### Predictive value of short-term outcomes evaluated with RECIST

The long-term prognosis was quite different from the short-term outcome evaluated with RECIST, with a sensitivity of 71.43% and a specificity of 44.44% in our study. Vincens *et al*.^17^ have reported that conventional MR imaging after treatment (median interval: 35 days) shows a sensitivity of 80% and a specificity of 55% in predicting residual disease of cervical cancers treated with CCRT, a result in line with our findings. Therefore, the short-term outcome evaluated with RECIST had a high false positive rate and a moderate false negative rate in predicting long-term outcomes for patients with cervical cancers treated with CCRT. This might because the radiation changes (inflammation, edema, and capillary hypervascularity) during the therapy may misleading the accurate differentiation between residual tumor and therapy induced changes.

### Predictive ability of tumour size

Larger pretreatment tumour sizes are considered to be related to poorer prognosis in patients with cervical cancers^5, 18^. However, we did not detect any significant difference of baseline maximum diameter of the lesion between patients with good and poor prognoses in this study, possibly because of the small sample size. Nevertheless, the pretreatment index contained little information regarding the therapeutic response; hence, its predictive value was compromised for evaluating patients undergoing treatment.

After the therapy started, the tumour size of the cervical cancer continued to decrease, and the good responders showed a greater rate of decrease than the poor responders. At time point 3 (4 weeks after the initiation of CCRT), an atrophy rate of maximum diameter over 58.31% proved effective in predicting a good long-term prognosis with an AUC of 0.751. Mayr *et al*. have reported similar results, in that tumours with fast regression rate during mid-therapy have the best rates of local control and disease-free survival in patients with cervical cancers^19^. However, evaluation of the tumour size was less effective and provided less rapid results than the IVIM parameters, as discussed below.

### Predictive ability of the *f* value

At time point 3, a lower *f* value of cervical cancer predicted poor prognosis with an AUC of 0.820. The *f* value is known as the vascular volume fraction of the voxel. A lower *f* value might indicate lower blood supply and hypoxic status of the tumour tissues, which are involved in local treatment failure after radiation therapy, a high incidence of regional or distant metastases, and poor disease-free survival rates in multiple solid tumours, including cervical cancer^20–23^. However, Hauser *et al*.^24^ have reported that a high initial *f* value is related to poor prognosis in patients with head and neck squamous cell carcinomas treated with radiotherapy in combination with chemotherapy and/or immunotherapy, possibly because of the different tumour types included in our study. In addition, Christine *et al*. and Yuh *et al*. have reported that cervical cancers with low perfusion derived by DCE MR imaging are likely to be resistant to radiation treatment as well as to have a high probability of developing metastases^25, 26^; these results may indirectly support our findings.

### Predictive value of diffusion related parameters

The ADC_IVIM_, D and ADC_DWI_ values of cervical cancer continued to increase during CCRT, possibly because of the decrease in cellular density and destruction of cellular membranes after effective treatment. At time points 2 and 3, a high ADC_IVIM_ value of cervical cancer predicted a good prognosis, with an AUC of 0.767 and 0.857, respectively. Somoye *et al*. have reported similar results in a study of patients with cervical cancers, in which survivors had a higher mid-treatment value (2 weeks following the therapy) (1.55 × 10^−3^ mm^2^/s) than non-survivors (1.36 × 10^−3^ mm^2^/s)^14^. Moreover, in the same study, a mid-treatment ADC value less than 1.40 × 10^−3^ mm^2^/s has been found to be associated with adverse outcomes, similarly to our results (cut-off value of ADC_IVIM_ at time point 2, 1.29 × 10^−3^ mm^2^/s). However, the pure diffusion coefficient D value demonstrated no significant power, thus indicating that perfusion effects might be an essential part of predicting treatment response. Moreover, the ADC_DWI_ value also was unable to predict long-term prognosis, probably because of the measurement error, owing to the limited number of *b* values.

According to our results, the IVIM sequence provided more diversified as well as more powerful parameters in predicting the long-term prognosis of advanced cervical cancer at early stages of CCRT. The IVIM parameters allow for earlier (two weeks after the initiation of CCRT) and more accurate (AUC of 0.857) prediction of long-term prognosis. The greater performance of IVIM may be a result of the multifunctional information that it provides. Furthermore, the IVIM parameters showed a significant positive relationship with different follow-up time points, but the age and FIGO stage of patients had weak associations with the parameters. Thus, IVIM parameters provide a good indication of the therapy-induced changes in tissue, which is more related to the treatment outcome than to the baseline clinical characteristics of the tumour.

## Limitations

There are several limitations in this study. First, the sample size was relatively small, and the pathological type of cervical cancers was simple (all squamous cell carcinomas) and hence might not be broadly representative. But every patient in our study received 3 MR examinations leading to a triple data size, which can help reduce the bias. Second, the follow-up time was relatively short, and longer follow ups are needed for confirmation. Third, multiple *b* values (14, 0∼1000 s/mm^2^) were arbitrarily selected for IVIM imaging, which required some technical optimization. Fourth, for several good responders, there was distinct size shrinkage, which made it difficult to exactly define the ROI. All the above issues require further investigation.

## Conclusion

IVIM-derived parameters have great potential for predicting long-term outcomes in patients with cervical cancers treated with CCRT. These parameters were superior to tumour morphologic information in predictive value. As a noninvasive technology that does not use exogenous contrast agents, IVIM MR imaging may be used as a routine radiologic procedure in clinical practice. If the patients at higher risk can be identified early, they could be offered treatment intensification, thus improving long-term survival.

## Materials and Methods

### Patients

This study was approved by the ethics committee of the Institutional Review Board of Nanjing Drum Tower Hospital, and written informed consent was obtained from all patients. All experiments were performed in accordance with relevant guidelines and regulations. Patients with histologically proven untreated cervical cancer (FIGO IIA ∼ IVB) who were scheduled to undergo CCRT, were prospectively included. The exclusion criteria were age below 18 years and contraindications for MR scanning (such as pacemaker implantation, artificial cochlea, or claustrophobia disorder) or CCRT (such as pregnancy, severe hepatic and renal dysfunction, or drug allergy).

From January 2014 to August 2016, 30 consecutive female patients with histologically confirmed uterine cervical cancers (all squamous cell carcinomas) were prospectively included. The median age was 51.1 years (range: 24∼76 years), and detailed characteristics of the patients are listed in Table 2.

All patients were treated with CCRT consisting of five weeks of external beam radiotherapy (EBRT) at a dose of 1.8∼2.0 Gy daily to a total dose of approximately 45 Gy and 3 weeks’ intracavitary brachytherapy with a total dose of 30∼40 Gy at point A. The definition of point A followed the American Brachytherapy Society consensus guidelines for locally advanced carcinoma of the cervix^27^. In addition, weekly nedaplatin or bi-weekly nedaplatin plus paclitaxel/docetaxel was concomitantly administered. The therapeutic regimens were adjusted according to the health conditions of individual patients.

### MR examination

All patients underwent MR examination three times: within 2 weeks before CCRT (time point 1), 2 weeks after the initiation of CCRT (time point 2), and 4 weeks after the initiation of CCRT (time point 3). All MR examinations were performed on a 3.0 T MR scanner (Achieva 3.0 T, Philips Healthcare, Best, the Netherlands) with a 16-channel phased array torso coil. The MR scan protocol was kept identical each time as follows: axial T2-weighted turbo spin-echo sequence (TR = 4,500 ms, TE = 90 ms, matrix size = 308 × 402, field of view = 30 cm × 40 cm, slice thickness = 5 mm, intersection gap = 0.5 mm, number of signal averages (NSA) = 1), sagittal T2-weighted turbo spin-echo sequence (TR = 4,500 ms, TE = 90 ms, matrix size = 212 × 209, field of view = 30 cm × 40 cm, slice thickness = 5 mm, intersection gap = 0.5 mm, NSA = 1), axial DW imaging sequence (TR = 4000 ms, TE = 238 ms, matrix size = 140 × 140, field of view = 25 cm × 25 cm, slice thickness = 5 mm, intersection gap = 0.5 mm, NSA = 1) with two *b* values of 0 and 800 s /mm^2^, axial IVIM sequence (TR = 2,834 ms, TE = 105 ms, matrix size = 152 × 120, field of view = 30 cm × 40 cm, slice thickness = 6 mm, intersection gap = 0.5 mm, NSA = 1, 14 *b* values = 0, 10, 20, 30, 40, 50,100, 150, 200, 350, 500, 650, 800, 1000 s/mm^2^), and axial contrast enhanced-T1 high resolution isotropic volume examination (e-THRIVE) (TR = 3.0 ms, TE = 1.42 ms, matrix size = 256 × 194, field of view = 30 cm × 40 cm, slice thickness = 1.5 mm, intersection gap = 0 mm, NSA = 1). Injection of 0.2 mL/kg bodyweight Gadodiamide (Omniscan, GE Healthcare, Shanghai, China) at a rate of 3.0 mL/s was followed by a 15 mL saline flush with a high pressure injector (Medrad Spectris Solaris EP MR Injector System; One Medrad Drive Indianola, PA, US). All MR examinations were performed with free breathing covering the whole pelvis. The scan time of IVIM sequence was approximately 10 min, and the total MR scan time was approximately 35 min. All the patients underwent three MR examinations successfully without any discomfort or side effects.

### Post-process and image analysis

Two radiologists (J.H. and ZY.Z) with 8 and 12 years’ experience in gynaecologic imaging independently performed post-process and image analysis. Cervical cancers showed hyperintense on T2 weighted, DW and IVIM images with high contrast enhancement. The maximum diameter of cervical cancer was measured with an axial T2 weighted image showing the largest area of the tumour referring to other MR sequences. The separate measurements of two radiologists were averaged to obtain the final results.

The axial DW imaging sequence, obtained with 2 *b* values (0 and 800 s/mm^2^), was loaded into the software (Extended MR Workspace 2.6.3.4; Philips Medical Systems, Best, the Netherlands). A mono-exponential model using two *b* values was used to calculate the ADC_DWI_ value^28^:

1 $${\rm{S}}/{{\rm{S}}}_{0}=\exp (-{b}^{\ast }{\rm{ADC}})$$

where S_0_ represents the signal without diffusion weighting, and S represents the signal with diffusion weighting.

The IVIM sequence was loaded into the software DWI-TOOL developed on the basis of IDL 6.3 (ITT Visual Information Solutions, Boulder, CO). A mono-exponential model using all 14 *b* values was used to calculate ADC_IVIM_ value by using the same equation as above^28^.

A bi-exponential model was applied to calculate D, *f*, and D* values with the following function introduced by Le Bihan *et al*.^15^:

2 $${{\rm{S}}}_{{\rm{b}}}/{{\rm{S}}}_{0}=(1-f)\times \exp (-b\times {\rm{D}})+f\times \exp (-b\times {{\rm{D}}}^{\ast })$$

where S_b_ represents the mean signal intensity with diffusion gradient *b*, and S_0_ represents the mean signal intensity in pixels without diffusion gradient. *f* is the microvascular volume fraction, D is the pure diffusion coefficient, and D* is the perfusion-related incoherent microcirculation. Considering that D* is much greater than D, the effects of D* on the signal decay at large *b* values (>200 s/mm^2^) can be ignored. Thus, at higher *b* values, Eq. (2) can be simplified into a Linear fit equation by which D can be estimated:

3 $${{\rm{S}}}_{{\rm{b}}}/{{\rm{S}}}_{0}=\exp (-{b}^{\ast }{\rm{D}})$$

Based on the value of D calculated using Eq. (3), *f* and D* values can then be calculated by using a partially constrained nonlinear regression algorithm based on Eq. (2).

With reference to other MR sequences, the axial DW or IVIM image that manifested the largest area of the tumour was selected, as well as the two adjacent slices above and below it. Regions of interest (ROIs) were manually drawn to cover as large an area of the solid part of the tumour as possible (mean: 934.12 mm^2^, range: 86.52∼2489.30 mm^2^), carefully excluding the macroscopic necroses, large vessels and areas with motion or magnetic susceptibility artefacts. The ROIs were copied to exactly the same locations on the ADC_DWI_, ADC_IVIM_, D, *f* and D* maps and the mean value from each ROI was obtained. The average value of the three slices and mean values from the two radiologists were calculated as the final results. If no residual tumour was observed after the treatment, ROIs were placed in the region where the lesion was initially located, and the maximum diameter was recorded as 0 cm. The change rate of a certain parameter was determined as: (time point A vs. B) = (value_B_ − value_A_)/value_A_ × 100%.

### Short-term and long-term outcome assessment

The short-term outcome was assessed one month after conclusion of the entire course of therapy by using morphological MR scanning according to RECIST as follows: (1) complete response (CR) was concluded if no residual tumour could be seen on the MR images; (2) partial response (PR) was concluded if the largest diameter of the residual tumour was 30% less than the original size or more; (3) progress of disease (PD) was concluded if there was an at least 20% increase in the longest diameter of the tumour compared with the pretreatment size; (4) the disease was considered as stable (SD) if there was neither a decrease sufficient to qualify for PR nor an increase sufficient to qualify for PD. According to the above criteria, 20 patients were classified as CR, and the remaining 10 were classified as PR; no PD or SD were defined.

Follow up was performed 1 month and 3 months after conclusion of CCRT and every 6 months thereafter until tumour recurrence or death. The long-term treatment outcome depended on the nearest follow-up with clinical (physical examination or pap smear) or imaging (CT and/or MR imaging) evidence: patients with complete remission was defined as having good prognosis, and patients whose outcomes were death, recurrence or sustained disease (patients classified as PR both after short-term and long-term follow-up) were defined as having poor prognosis. During a median follow up of 24 months (range: 10∼34 months), 21 patients had a good prognosis whereas the remaining 9 patients had a poor prognosis (5 deaths, 3 local recurrences, and 1 sustained disease) (Table 2).

### Statistical analyses

Statistical analyses were performed with SPSS 16.0 (SPSS Inc., Chicago, IL). All quantitative values in normal distribution are presented as the mean ± standard deviation (SD). Multiple linear regression was used to assess interactions between clinical characteristics and the IVIM parameters. Repeated measurements analysis of variance (ANOVA) was used for the overall differences of each parameter between different time points as well as different groups. A paired samples *t*-test was performed to detect significant changes in IVIM-related parameters and tumour size changes during CCRT. A chi-square test was performed to determine the relationship between the short-term outcome and the long-term treatment outcome, and the relationship between different FIGO stages and the long-term treatment outcome. Receiver operating characteristic (ROC) analysis was performed for the predictive value of parameters with significant differences between different prognoses. The intraclass correlation coefficient (ICC) was calculated to evaluate interobserver agreement in the measurement of IVIM-related parameters. Two-tailed *p* values less than 0.05 were considered statistically significant.

### Data Availability

The datasets generated during and/or analysed during the current study are available from the corresponding author on reasonable request.
